# Moving beyond individual choice in policies to reduce health inequalities: the integration of dynamic with individual explanations

**DOI:** 10.1093/pubmed/fdy045

**Published:** 2018-03-13

**Authors:** N M Kriznik, A L Kinmonth, T Ling, M P Kelly

**Affiliations:** 1 THIS Institute (The Healthcare Improvement Studies Institute), University of Cambridge, Cambridge Biomedical Campus, Clifford Allbutt Building, Cambridge, UK; 2 Department of Public Health and Primary Care, Cambridge Institute of Public Health, University of Cambridge, Forvie Site, Robinson Way, Cambridge, UK; 3 RAND Europe, Westbrook Centre/Milton Rd, Cambridge, UK

**Keywords:** dynamic and relational explanations, health inequalities, individualistic explanations

## Abstract

**Background:**

A strong focus on individual choice and behaviour informs interventions designed to reduce health inequalities in the UK. We review evidence for wider mechanisms from a range of disciplines, demonstrate that they are not yet impacting on programmes, and argue for their systematic inclusion in policy and research.

**Methods:**

We identified potential mechanisms relevant to health inequalities and their amelioration from different disciplines and analysed six policy documents published between 1976 and 2010 using Bacchi’s ‘What’s the problem represented to be?’ framework for policy analysis.

**Results:**

We found substantial evidence of supra-individualistic and relational mechanisms relevant to health inequalities from sociology, history, biology, neuroscience, philosophy and psychology. Policy documents sometimes expressed these mechanisms in policy rhetoric but rarely in policy recommendations, which continue to focus on individual behaviour.

**Discussion:**

Current evidence points to the potential of systematically applying broader thinking about causal mechanisms, beyond individual choice and responsibility, to the design, implementation and evaluation of policies to reduce health inequalities. We provide a set of questions designed to enable critique of policy discussions and programmes to ensure that these wider mechanisms are considered.

## Introduction

Current UK policies designed to reduce health inequalities through preventing non-communicable disease are based largely on an individualistic epistemology. The dominant epistemic assumption (i.e. the assumption about what constitutes admissible evidence to guide action) in the implementation of public health programmes is that human behaviour is a major determinant of health, and that behaviour is largely a matter of individual choice; individuals are therefore responsible for their own health and for making health-related behaviour changes. Reliance on such simple, linear causal explanations and of proximal risk factors in the causes of disease has consistently led to a focus on changing individual behaviour.^1–6^

We argue for a greater consideration of how more complex relational and dynamic factors, beyond just the individual, impact on health. A number of disciplines including sociology,^7^ history,^8^ biology,^9^ neuroscience,^10^ philosophy^11^ and psychology^12^ have produced evidence of potential mechanisms. The existence of interactions with place and social context, power, economics, institutional relationships and biology, over time and across generations have been widely cited.^2,13–18^ Developments in biology include epigenetics^9^ and the neuropsychology of executive functioning;^12^ and both are highly relevant to understanding how health inequalities are sustained. These remain largely outside the purview of current policy interest.

While some of this evidence may sometimes find its way into policy statements it is seldom realized in interventions.^3,19,20^ The six documents chosen for analysis in the study demonstrate that the main assumptions underpinning interventions to address health inequalities have not changed significantly since the mid-1970s, and remain focussed on individual responsibility. The policy interventions put forward reflect this partial view of the totality of the evidence about the generation and amelioration of health inequalities and the social patterning of disease. We highlight this constancy and propose initial steps to change the dominant view with an alternative approach.

We argue that the adoption of a dynamic, relational epistemology is fundamental to a fuller realization of causal mechanisms and for the design and implementation of more effective interventions to address health inequalities. The success of tobacco control in the UK stems from its base in the dynamic and relational aspects of practices linked to tobacco consumption. This includes, as well as individual choices, both the role of markets and advertising, and explicit action to counteract ‘dirty tricks’ of the tobacco industry over time. By dynamic we refer to changes over time, and by relational we mean the need to consider the relationships involved in the practice of smoking which go beyond the individual just physically smoking a cigarette.

Three themes are central to this approach: ‘power’, in exploring the relations between groups and between groups and institutions in society; ‘history’, in investigating how relations change or are sustained over time; and the dynamic ‘relationship between the biological and the social’. We explore these themes below.

We first identify evidence of the importance of relational and dynamic factors in understanding the problem of health inequalities. Second, we examine policy approaches over several decades concerning health inequalities, demonstrating the repeated focus on individual behaviour change. Finally, we develop a set of questions which highlight this broader perspective, to sensitize policy-makers, service developers and researchers when developing policies to address health inequalities.

## Moving towards more dynamic thinking: power, history and the relationship between the biological and the social

### Power

Health inequalities are seldom described in policy documents in terms of power relations and competition for scarce resources between classes, genders and ethnic groups, or the mechanistic consequences of these power relations on biology. The idea of ‘empowering’ the individual to make healthier choices is a central tenet of most policy proposals, but discussions of the impact of power dynamics between groups and institutions are overlooked, underplayed or ignored. The interactions and intersections between different groups are essential to the production of social structures and forms of inequality. Power is a central part of these interactions, but individualistic forms of analysis are poor at elucidating such dynamics. The focus on individual lifestyles, for example, precludes any meaningful analysis of the power dynamics in which individuals are involved and that, in turn, influence the types of choices they make.

Power relations are intrinsic to social life.^21^ At their most basic, power relations affect people’s access to resources, including health services, and determine the lived experience of discrimination, disadvantage, bullying, harassment and social exclusion. They influence educational and employment opportunities, including the type of work people are able to do and their contracts of employment. The associations between these things and their links to health inequalities have been known for decades^22^ but the mechanisms of their direct impacts on people’s lives have not featured in government policy documents. Of course, attempts to promote equality have been part of government policy but these have tended to focus on protected characteristics rather than on the nature of the damaging or health protecting nature of the relations themselves.^23^ Following Sen we should consider the degree to which health policy and its implementation enhance the capacity for health, for example.^24^

### The historical perspective

Likewise, history and a longer time perspective are largely absent from policy discussions of health inequalities. Insofar as history appears it is in terms of a progressive narrative about public health advances since the 19th century.^25–27^ There is a form of institutional memory loss evident in policy where similar ideas are rediscovered and recycled over a relatively short period of time,^20^ and policies demonstrate historical amnesia about the processes which have led to contemporary health inequalities. This leads to, for example, the assumption that health inequalities can be explained by current societal conditions alone and ignores endemic social arrangements which persist over decades and longer. It leads to overoptimistic expectations of performance of likely effective programmes, with rapid payback being expected and public health initiatives facing cuts if they do not produce results swiftly. An historical perspective in policy and research informs a clearer understanding of the longer-term intertwining causes, construction and maintenance of health inequalities. According to Raadschelders, all too often ‘History … is regarded as a ‘past’ that can be recorded for its own sake but has little relevance to contemporary challenges. This view of history is the product of a diminished and anemic sense of time, resulting from organizing the past as a series of events that inexorably lead up to the present in a linear fashion’.^28^ We argue that bringing a historical perspective provides a richer understanding of causation that recognizes the ‘layered’ nature of how the past leaves its mark on the ways health inequalities are reproduced over time. This perspective helps to illuminate the tools available to policy-makers and the interventions which could have a lasting impact.^29–30^

Social epidemiology has long shown recurring patterns of health disadvantage geographically lasting generations in many towns and cities in the UK. Notwithstanding policy efforts, these patterns have remained stubbornly fixed. Generation on generation, the consequences of the historical reproduction patterns of health differences remain significantly unchanged. It is no longer infectious disease that causes premature mortality in the poorer parts of the country, but non-communicable disease. Absolute death rates are lower than the 19th century but relative differences remain. Meanwhile policy is fixated on simple behavioural solutions not on the reasons why the structural differences are so intractable.^31^

### The biological and the social

Descriptions of the role of biology and its interplay with social factors in producing population patterns of disease, was associated originally with Engel^32,33^ and later with Barker.^34–37^ The relationship across generations between the environment and biological moderation of gene expression (epigenetics) is now providing new evidence of interacting social and biological mechanisms underlying the transgenerational transmission of inequalities in health,^2,38^ leading to a re-consideration of cross-generational effects on poor health and amelioration or exacerbation through social conditions. It suggests a re-emphasis on programmes to support maternal, foetal and child health as a key strategy in breaking the cycle of inequality in health.

## Policy

We now turn to an examination of existing policy approaches to addressing health inequalities. We review the extent to which evidence of the mechanisms considered above are informing policy recommendations.

We reviewed six key policy documents published by the UK government or the English Department of Health between 1976 and 2010^25,27,39–42^ using Bacchi’s ‘What’s the problem represented to be?’ (WPR) framework for policy analysis (Table 1).^43,44^ The interrogation of documents using this framework allows for comparisons over time as well as the development of an understanding of individual documents and the assumptions which underpin policy rhetoric. This examination demonstrated a longstanding continued focus on individual behaviour in strategies to reduce health inequalities, and more generally to prevent non-communicable disease (see Table 2 for verbatim quotations from the policy documents^45^).

**Table 1:** Bacchi’s^43,44^ ‘What’s the problem represented to be?’ framework for policy analysis

Questions in the WPR framework	Aim of question
1. What is the ‘problem’ represented to be in a specific policy?	To understand how a phenomenon comes to be understood as a problem in social policy (problematization), including the causes of the problem.
2. What presuppositions or assumptions underlie this representation of the ‘problem’?	To understand the discursive practices surrounding the representation of the problem (archaeology), i.e. what can and cannot be said about a problem.
3. How has this representation come about?	To understand the history (genealogy) of the development of understanding of a problem.
4. What is left unproblematic in this problem representation?	To identify silences and highlight explanations which are not discussed and to consider why these views might be excluded from this particular representation of the problem.
5. What effects are produced by this representation of the ‘problem’?	To understand the creation of subjectivities produced by representations of problems in policies: how individuals and population groups are conceptualized.
6. How/where is this representation of the ‘problem’ produced, disseminated and defended? How has it been (or could it be) questioned, disrupted and replaced?	To identify where this representation of the problem has been reproduced, including in other policy documents.

**Table 2:** Policy documents and their individualistic focus (Derived from Kriznik^23^, unpublished PhD thesis.)

Publication	A	B	C	D	E
	Focus on behaviours and choices	Focus on wider influences	Focus on proximal factors	Individual, group or population risk	Cause and effect explanations
1. Prevention and Health—Everybody’s Business (1976)	Many of the current major problems in prevention are related less to man’s outside environment than to his own personal behaviour; what might be termed our lifestyle (p. 17) Affluence is not an unqualified boon and while it has certainly enabled us to avoid some diseases, for example those due to nutritional deficiency, it has opened the door to others arising, for instance, from unwise behaviour and over-indulgence in one form or another (p. 31)	Technological developments in transport and communications, in industry, and in the production and marketing of food, are having an effect for better or worse on people’s health, whilst the physical environment itself is undergoing changes in a number of relevant ways (p. 31)	To a large extent though, it is clear that the weight of responsibility for his own state of health lies on the shoulders of the individual himself. The smoking related diseases, alcoholism and other drug dependencies, obesity and its consequences, and the sexually transmitted disease are among the preventable problems of our time and in relation to all of these the individual must choose for himself (p. 38)	The key to prevention is often the identification of ‘risk factors’ and thus of ‘vulnerable groups’. A risk factor is a characteristic of an individual which has been found to be statistically associated with a disease. Where such an association is known to exist between a characteristic and a disease, the persons possessing this characteristic are a vulnerable group (p. 92)	There is much potential for prevention in health education aimed at altering people’s attitudes towards such things as tobacco, alcohol and exercise—persuading them in effect to invest in their own health (p. 87)
2. The Health of the Nation: A Strategy for Health in England (1992)	On behaviour—lifestyles—a balance of action is needed. People cannot be forced to behave sensibly in terms of their smoking, eating, exercise, alcohol or personal sexual habits. But efforts can be made to ensure that when they choose, they are exercising informed choice in circumstances where this is possible. (par. 3.4)	A number of key strategic policy objectives and guiding principles underpin the entire approach. They are the need:…to recognize that as health is determined by a whole range of influences—from genetic inheritance, through personal behaviour, family and social circumstances to the physical and social environment—so opportunities and responsibilities for action to improve health are widely spread from individuals to Government as a whole. (par. 2.6) The reasons for these variations are complex. The Government does not believe there is any panacea—here or elsewhere in the world either in terms of a full explanation or a single action which will eradicate the problem. But neither difficulty is a reason for inertia. Progress can be made on three fronts: first, through the continued general pursuit of greater economic prosperity and social wellbeing; second, through trying to increase understanding of the variations, and the action which might effectively address them; third, through specific initiatives to address the health needs of particularly vulnerable groups, whether geographical, ethnic, occupational or others who need specific targeted help. (par. 4.15)	…there is considerable emphasis in this document on the need for people to change their behaviour— whether on smoking, alcohol consumption, exercise, diet, avoidance of accidents and, with AIDS, sexual behaviour. The reason is simple. We live in an age where many of these main causes of premature death and unnecessary disease are related to how we live our lives. (Foreword)	In framing action within key areas the needs of specific groups of people within the population must be considered; the particular needs of children, women, elderly people and people in black and ethnic minority groups and certain socio-economic groups are also considered in the appendix. (par. 2.15)	Government must ensure that individuals have the necessary information with which they can exercise informed free choice. Education is the key. Equally, Government undertakes a variety of measures designed to ensure that people live in physical and social circumstances where such free choice is possible. (Foreword) Everyone has a part to play in improving health… To seize the opportunity, people need information to help make the right choices. Reliable health education in its widest sense is essential for this—pervading education at school and also the many sources of information for people generally about health and its determinants. (par. 3.8) The reasons for these variations are by no means fully understood. They are likely to be the result of a complex interplay of genetic, biological, social, environmental, cultural and behavioural factors… In part they are accounted for by differences in risk behaviour. (Appendix F)
3. Saving Lives: Our Healthier Nation (1999)	The Government recognizes the importance of individuals making their own decisions about their own and their families’ health. But we also believe that there are steps we can take to help support the decisions people make. (par. 3.4)	Improving health means tackling the causes of poor health. We know that the causes of ill-health are many: a complex interaction between personal, social, economic and environmental factors. (par. 1.21) Individual behaviour is often vitally important in improving, safeguarding or damaging health. But poor health can also spring from a complex interaction between the genetic make-up and behaviour of individuals and social, economic and environmental factors in the community. (par. 4.1)	How people live their lives—what they eat, how active they are, whether they smoke—is central to improving health. Other factors, including people’s education, employment, housing and environment also play a key role. (par. 3.1)	Our modern approach is reflected in the goals of this White Paper: to improve the health of the population as a whole by increasing the length of people’s lives and the number of years people spend free from illness; and to improve the health of the worst off in society and to narrow the health gap. (par. 1.17) And while death rates are improving substantially for the best off in society, the worst off have not benefited to anything like the same extent, thus widening the health gap. (par. 6.1)	Government will play its part by creating the right conditions for individuals to make healthy decisions. Across a range of Government policy, we are focusing on the factors that increase the likelihood of poor health—poor housing, poverty, unemployment, crime, poor education and family breakdown. (par. 1.37) Every day people are faced with decisions in their daily lives, including decisions which affect their health. Sometimes they recognize that certain decisions put their health at greater risk than others. But it is not always clear how great or small a risk they are taking… We can help people to understand better about risk. (par. 3.15-3.16) In short, it is the role of the Government to provide information about risk. But in most cases it is for the individual to decide whether to take the risk. (par. 3.25)
	Ten Tips For Better Health Don’t smoke. If you can, stop. If you can’t, cut down. Follow a balanced diet with plenty of fruit and vegetables. Keep physically active. Manage stress by, for example, talking things through and making time to relax. If you drink alcohol, do so in moderation. Cover up in the sun, and protect children from sunburn. Practise safer sex. Take up cancer screening opportunities. Be safe on the roads: follow the Highway Code. Learn the First Aid ABC— airways, breathing, circulation.				
4. Tackling Health Inequalities: A Programme for Action (2003)	Individuals also have to be responsible for their own health and that of their children by making appropriate and informed lifestyle choices on smoking, diet and exercise, all of which can widen health inequalities. It is essential that such choices should be informed by clear and accurate advice. Schools have a vital part to play while charities and healthcare professionals, including community pharmacists and dentists, can advise how to quit smoking, offer exercise on prescription, identify patients at risk of heart disease and provide services for substance misusers. (par. 5.36)	Overall, health and life expectancy are still linked to social circumstances and childhood poverty. (par. 1.1) The Government’s aim is to reduce health inequalities by tackling the wider determinants of health inequalities, such as poverty, poor educational outcomes, worklessness, poor housing, homelessness and the problems of disadvantaged neighbourhoods. (par. 1.8) The Acheson inquiry report emphasized the need for effective interventions to address the wider influences on health inequalities. Government departments have contributed to progress in addressing these determinants, such as improving educational attainment and tackling low basic skills, improving the quality of poor housing, improving the accessibility, punctuality, reliability and use of local transport, tackling worklessness and inactivity, and improving access to social and community facilities and services. Regional Development Agencies (RDAs) have been set up to act as the strategic drivers of regional economic development. (par. 3.34)	The challenge, therefore, will be to ensure that future improvements in health over the next 20 years are shared by all. The widening health gap reflects current realities. Experience has shown that the potential to generate and share health gains across the population by preventive action—for example, by targeting smoking and sedentary lifestyles—has yet to be fully realized. So policies need to ensure that health gains are matched by a narrowing of the health gap. (par. 1.5)		The reasons for these differences in health are, in many cases, avoidable and unjust—a consequence of differences in opportunity, in access to services, and material resources, as well as differences in the lifestyle choices of individuals. Unfortunately, the effects can be passed on from generation to generation. (par. 1.4)
				Generally, the more affluent people are, the better will be their health; conversely, the poorer people are the worse will be their health. But there are wide differences among social groups. This Programme for Action does not, therefore, just address the most disadvantaged groups and areas. It also addresses the needs of a large part of the population as well as those of socially deprived groups. (par. 1.3)	
5. Choosing Health: Making Healthy Choices Easier (2004)	Choosing health sets out how we will work to provide more of the opportunities, support and information people want to enable them to choose health. It aims to inform and encourage people as individuals, and to help shape the commercial and cultural environment we live in so that it is easier to choose a healthy lifestyle. (Foreword by Tony Blair) Success in developing demand for health is not enough on its own; people need to be able to make informed choices about what action to take. (Chapter 2 par. 18)	People who are disabled or suffer from mental ill health, stretched for money, out of work, poorly qualified, or who live in inadequate or temporary accommodation or in an area of high crime, are likely to experience less control over their lives than others and are often are pressed to cope with immediate priorities. They are often less likely to think about the consequences of everyday choices about diet, exercise, smoking and sexual behaviour on their long-term health, or to take up the childhood immunization and health screening programmes that provide protection against diseases that can kill or cause serious long-term ill-health. (Chapter 1 par. 17)	The choices people make as consumers—what we eat and drink, and how we use services and facilities—impact on health. (Chapter 2)	Many of the initiatives in this White Paper will be targeted first at communities and groups where opportunities to choose health are least well-developed and most progress is needed. (Chapter 1 par. 20) We also need to look at ways to make healthy choices more accessible to individuals and groups who may not find it easy to use information designed to meet the needs of the general population. (Chapter 2 par. 35)	It is a fact of life that it is easier for some people to make healthy choices than others. Existing health inequalities show that opting for a healthy lifestyle is easier for some people than others… The success of the strategy will be measured first in the increased number of healthy choices that individuals make, and then in the lives saved, lengthened and improved in quality. (Preface by John Reid, Health Secretary) The new approaches set out in this chapter will help people by offering them the opportunity to develop their own personal health guides and providing access to NHS-accredited health trainers and other NHS and community resources to support them in acting on their plans for health. (Chapter 5 par. 37)
6. Healthy Lives, Healthy People (2010)	We need a new approach that empowers individuals to make healthy choices and gives communities the tools to address their own, particular needs. (Foreword)	We are all strongly influenced by the people around us, our families, the communities we live in and social norms. Our social and cognitive development, self-esteem, confidence, personal resilience and wellbeing are affected by a wide range of influences throughout life, such as the environment we live in, the place in which we work and our local community. This impacts on our health and our life chances. (par. 1.13) Wider factors that shape the health and wellbeing of individuals, families and local communities—such as education, employment and the environment—also need to be addressed in order to tackle health inequalities. (par. 2.4)	Our causes of premature death are dominated by ‘diseases of lifestyle’, where smoking, unhealthy diet, excess alcohol consumption and sedentary lifestyles are contributory factors. (par. 1.2)	When it comes to improving people’s health and wellbeing, we need a different approach. We cannot just ban everything, lecture people or deliver initiatives to the public. This is not justified and will not work. Nor should we have one-size-fits-all policies that often leave the poorest in our society to struggle. (par. 2.28) This includes changing social norms and default options so that healthier choices are easier for people to make. There is significant scope to use approaches that harness the latest techniques of behavioural science to do this—nudging people in the right direction rather than banning or significantly restricting their choices. (par. 2.34) To meet the challenges set out in earlier chapters, the Secretary of State for Health intends to create a new public health system in England to protect and improve the public’s health, improving the health of the poorest, fastest. (par 4.1)	Our health and wellbeing is influenced by a wide range of factors—social, cultural, economic, psychological and environmental—across our lives. These change as we progress through the key transition points in life—from infancy and childhood, through our teenage years, to adulthood, working life, retirement and the end of life. Even before conception and through pregnancy, social, biological and genetic factors accumulate to influence the health of the baby. (par. 1.12)

Successive documents did show increasing reference to the impact of a wide range of factors on health (social, cultural, economic and environmental). Nevertheless, these were rarely then used for policy and intervention development or for the evaluation of programmes; in both, individualism remained paramount. For example, ‘Saving Lives: Our Healthier Nation’ argued that ‘the causes of ill-health are many: a complex interaction between personal, social, economic and environmental factors’ (Table 2, 3B). Yet the main approach offered to reducing health inequalities focused on ensuring individuals are informed about risks to their health as ‘in most cases it is for the individual to decide whether to take the risk’ (Table 2, 3E). The responsibility falls to the individual to make the least or less risky decision. Here and elsewhere there is a ‘disconnect’ between policy rhetoric about causes and recommended remedial actions.

Evidence about the wider determinants of health is used to justify interventions on health inequalities, which then paradoxically concentrate on individual behaviour. Rather than addressing the wider determinants themselves, certain groups are viewed as lacking the capacity to negotiate successfully the effects of these determinants, demonstrated by their proclivity for making unhealthy choices. Thus, a number of interventions focus on the provision of opportunities to enable individuals to improve their current social and economic situation through, for example, education, training and work opportunities.^41^ A common argument is that by embracing such opportunities people will have more control over their lives, which would result in them being more interested in their health and therefore more likely to make healthy choices.^42^ The idea of the individual as a chooser dominates these discussions.^45^ This idea shapes the understanding of the problem as one of individuals, in themselves, being incapable of making healthy choices unless steered by interventions which change their attributes as individuals who will then change their behaviours. The result has been described as ‘lifestyle drift’^46^ in policy measures and ‘lifestyle push’ from politicians and markets.^20^

This focus on the individual effectively neutralizes the effects of social context and airbrushes out of the picture a number of important contextual agents and institutions—specifically the state, markets and industry. So the state and its retreat from interventionism consequent on neo-liberal economic thinking, the role of markets (as a cause of rather than a solution to the problem), and the (incidental) health damaging roles of the food, advertising and alcohol industries are conveniently put to one side.^47^ Actions addressing these actors and getting them to change are not central to contemporary policy focus. The alternative view that the State has a duty to enable as far as possible everyone to have a fair opportunity to live a healthy life and that governments should try to remove inequalities that affect disadvantaged groups or individuals, including a duty of proportionate regulation, has not always been a dominant motif in policy documents.^23,48^ Following Sen, among others, we would emphasize the importance of addressing how public and private actions and organizations shape the capability of individuals to make positive choices regarding their health.^49^

For example, obesity as a significant public health threat and an important cause of health inequalities has become engrained in policy discourse in the last decade and a half.^19^ Numerous policy documents and a raft of guidance have been produced on the topic, including the recent 2016 childhood obesity strategy.^19,50,51^ Yet success in curbing obesity has been minimal. This is despite compelling evidence that an obesogenic environment is generating the obesity epidemic,^15,50^ and that its structure and dynamics should be the target for arresting the epidemic. Policy solutions have persistently focused on proximal determinants, most prominently individual diet and exercise.^52^ Similarly with alcohol consumption, the focus remains on individual capability and how to ensure individuals make healthy choices,^4,53^ reinforced by statements by ministers past and present.^54^

A most important exception to this perspective was the Foresight report on ‘Tackling Obesities’, commissioned by the Government Office for Science and published in 2007. The report presented evidence demonstrating the social and biological complexity of obesity, and emphasized the need to intervene beyond individuals into processes of governance and decision-making to stem rising obesity and the necessity of evaluation: ‘The deceptively simple issue of encouraging physical activity and modifying dietary habits, in reality, raises complex social and economic questions about the need to reshape public policy in food production, food manufacturing, healthcare, retail, education, culture and trade’.^15^ The mid-term review of the Report at 3 years drew attention to ‘stakeholder inertia in adopting more accurately informed perspectives of the roles of the individual and of society (which) can hinder the development of strategies and interventions to manage the current and future obesity epidemic’. Implementation of the report was terminated long before effects could be appropriately evaluated.

There are a small number of other examples where what we refer to as a relational approach has found its way into the public domain. For example the NICE Public Health Guidelines on Community Engagement,^55^ Social and Emotional Wellbeing of Children,^56,57^ Healthy Working Conditions,^58^ Preventing Harmful Drinking^59^ and Preventing Cardiovascular Disease.^60^ Not only were these exceptions, but also in the case of the Cardiovascular Disease and Harmful Drinking guidelines they were rejected by Ministers. One of the implications of the focus on individuals and on behaviour change is that it pushes policy interest towards proximal risk factors (which the NICE Guidelines did not) and the role of these in the aetiology of non-communicable disease in particular. However, knowledge about risk and its links to behaviour in causal pathways of disease do not on their own provide any solutions as to how to change those things. For example, knowledge about the dangers of exposure to cigarette smoke or alcohol suggests that reducing exposure would be beneficial, but does not explain how to achieve that. A focus on the dynamics of the systems involved in the relations between industry, markets, advertising, human preferences, group behaviour, as well as the individual actor provides a richer theoretical frame for developing and evaluating integrated programmes to address the problem. This has been the case with the success of tobacco control in the UK. We advocate a similar ‘how to’ approach with respect to obesity, alcohol and physical activity.^31^

## Discussion

### Main finding of this study

We have highlighted extensive cross-disciplinary evidence about the relational nature of health inequalities, and causal mechanisms beyond individual choice and responsibility. We have shown that this evidence rarely follows through to preventive programmes.

### What is already known on this topic

Health inequalities persist in the UK driven largely by the social patterning of non-communicable disease. Interventions designed to reduce health inequalities are still primarily shaped by a focus on individual behaviour. Yet, there is substantial evidence of supra-individualistic and relational mechanisms relevant to health inequalities from a range of disciplines including sociology, history, biology, neuroscience, philosophy and psychology. This evidence is not yet applied systematically in policy or action, where it may inform the design and implementation of more effective policies.

### What this study adds

A perspective recognizing the complexity of the systems in which public health must practice, including its politics, shifts us away from narrowly focussed linear behaviour change models to a focus on reflexive systems and the power of players in those systems.^61–65^ Prioritizing the study of relationships between people and groups of people, how these relationships are sustained and change over time, and acknowledging links between power, time and the biological and social, we suggest will bring into the discourse a wider perspective to complement the existing focus on individual behaviour change.

In order to move towards integrating this thinking into policy considerations, we have developed a set of questions to use in writing and critiquing policy papers, which aim to ensure that proposed interventions to address health inequalities take into account relational and dynamic factors, as well as individual behaviour (Table 3). These questions can be used not only by policy-makers and service developers but also by academic researchers to ensure that relational and dynamic factors are brought to the forefront of policy evaluation.

**Table 3:** Questions to use in the formulation and critique of policies to address health inequalities

Questions to consider when developing policy recommendations to address health inequality	Aim of questions
*Inclusion of wider determinants*	
1.Are proximal risk factors used as the primary justification for solutions to address health inequalities?	To highlight the type of evidence being used to justify solutions and to identify any gaps particularly around wider determinants of health.
2.Is evidence included relating to the influence of the wider determinants of health?	
*Evidence over time*	
3.Have the recommended approaches to addressing health inequalities appeared in policy documents in the last 2, 5, 10 and 15 years?	To consider previous attempts to address health inequalities; to highlight that this is a problem with a long history rather than a contemporary issue; and to draw attention to both evidence of effect/non-effect and lack of testing over time.
4.Have these approaches shown cost-effectiveness in formal studies over sufficient time intervals?	
*Causal pathways and mechanisms of action*	
5.Are there clear steps from identification of a cause of the problem to actionable interventions?	To ensure that factors listed as contributing to health inequalities are adequately addressed through causal pathways. Policies should include a guide to implementation of interventions in order to move from rhetoric to action.
6.Are the mechanisms of action of the recommended intervention described?	
*Social context and power*	
7.Are the recommendations grounded in the social and economic contexts of everyday life?	To draw attention to the importance of social context in enabling or restricting change, and to the nature of power.
8.How are the relationships between the state, industry, civil society and individuals taken account of in explanations for health inequality and proposals for action?	
*History and biology*	
9.What evidence of historical social conditions have been used in the analysis?	To emphasise the importance of the dynamism of the problem of health inequalities from a historical perspective; and to acknowledge the interface between the social and the biological.
10.Are there any considerations of the relationships between social and biological processes?	

### Limitations of this study

This is not a comprehensive or systematic review of all documents relating to English policy recommendations to address health inequalities.

## Conclusion

Current policies dominating efforts to reduce health inequalities through prevention of non-communicable disease target individual behaviour change and have not worked well. We have argued that an individualistic epistemology limits their impact. Programmes predominantly focus on individual behaviour change foregrounding individual choice and responsibility. There exists strong and extensive evidence that interconnecting and interacting factors, beyond the individual, impact on health including place, context, power, economics, institutional relationships and biology, over time and across generations. The existence of such evidence, however, has not been sufficient to garner policy action even when existing strategies have failed to successfully address health inequalities. We propose a list of questions that researchers and policy-makers can use when writing or critiquing policy in order to bring this broader perspective to the forefront of their analysis of the problem. This is one small step in moving from the rhetoric of whole systems interventions to long-term intervention and evaluation and towards broadening the range of approaches and evidence we use to unpack the problems of health inequalities and work towards policies to address them.
